# Fabrication and Characterization of Diclofenac Sodium Loaded Hydrogels of Sodium Alginate as Sustained Release Carrier

**DOI:** 10.3390/gels7010010

**Published:** 2021-01-27

**Authors:** Muhammad Suhail, Arshad Khan, Jessica M Rosenholm, Muhammad Usman Minhas, Pao-Chu Wu

**Affiliations:** 1 School of Pharmacy, Kaohsiung Medical University, 100 Shih-Chuan 1st Road, Kaohsiung City 80708, Taiwan; Suhailpharmacist26@gmail.com; 2 Department of Pharmaceutics, Faculty of Pharmacy, Khawaja Fareed Campus (Railway Road), The Islamia University of Bahawalpur, Punjab 63100, Pakistan; arshadpharma77@gmail.com; 3 Pharmaceutical Sciences Laboratory, Faculty of Science & Engineering, Åbo Akademi University, BioCity (3rd floor), Tykistökatu 6A, 20520 Turku, Finland; jerosenh@abo.fi; 4 College of Pharmacy, University of Sargodha, Sargodha 40100, Pakistan; 5 Department of Medical Research, School of Pharmacy, Kaohsiung Medical University Hospital, Kaohsiung 80708, Taiwan; 6 Drug Development and Value Creation Research Center, Kaohsiung Medical University, Kaohsiung 80708, Taiwan

**Keywords:** hydrogels, sodium alginate, in-vitro study, dissolution, kinetic modeling

## Abstract

The aim of the current study was to fabricate naturally derived polymer based hydrogels for controlled release of diclofenac sodium (DS) for a long duration of time. In this research work, sodium alginate-co-poly(2-acrylamido-2-methyl propane sulphonic acid) (SA-co-poly(AMPS)) hydrogels were prepared by the free radical polymerization technique, where sodium alginate (SA) and 2-acrylamido-2-methyl propane sulphonic acid (AMPS) were used as the polymer and monomer while ammonium peroxodisulfate (APS) and N,N′-Methylene bisacrylamide (MBA) were used as the initiator and cross-linker, respectively. A swelling study was performed to determine the swelling index of developed hydrogels in both acidic (pH 1.2) and basic (pH 7.4) media and pH-independent swelling was observed due to the presence of AMPS. An in vitro release study was conducted to evaluate the percentage of drug released, and a high release of the drug was found at the higher pH of 7.4. Sol–gel analysis was performed to analyze the crosslinked and uncrosslinked part of the hydrogels, and results showed a rise in gel fraction as the composition of SA, AMPS and MBA increased while the sol fraction decreased and vice versa. This work demonstrated a potential for sustained delivery of diclofenac sodium by employing various concentration of SA, AMPS and MBA.

## 1. Introduction

Nonsteroidal anti-inflammatory drugs (NSAIDs) are a class of drug which decrease pain, reduce fever, stop the clotting of blood and reduce inflammation in higher doses [1,2]. An enzyme, cyclooxygenase (COX) plays an important role in the biosynthesis of prostaglandins. NSAIDs inhibit the COX enzymes, due to which biosynthesis of prostaglandins is stopped and thus, NSAIDs exert their anti-inflammatory effect in this way [3].

DS (an NSAID) is the sodium salt of o-(2,6-dichlorophenylamino)-phenyl acetic acid. It is used in management of ankylosing spondylitis, rheumatoid arthritis and osteoarthritis because it has antipyretic, analgesic and anti-inflammatory pharmacological effects. Currently DS is available in market as topical gels, tablets and capsules. The recommended dosing regimen of DS as tablets is 25, 50, 75 and 100 mg, while as capsules it is 18 and 35 mg respectively for two, three or four times in a day. The half-life of DS is very short (1–2 h). It is absorbed rapidly and completely when administered orally. Hence taking of DS two, three or four times in a day results in undesirable effects such as peptic ulcers and gastrointestinal bleeding associated with repeated dosing of DS in the management of diseases [4,5,6]. Therefore, new strategies for delivery of DS are needed to overcome the adverse effects associated with rapid administration. Due to this reason, we prepared sodium alginate based hydrogels to prolong the release of DS and reduce the problems such as gastrointestinal bleeding and peptic ulcers.

Hydrogel is a network of crosslinked hydrophilic polymer chains that has the capability to accommodate a high quantity of water due to its hydrophilic nature [7]. Due to this unique property, hydrogels are used for different purposes especially in tissue engineering, the pharmaceutical industries and the biomedical field. Besides this, hydrogels also play an important role in the development of drug delivery systems [8,9,10]. High biocompatibility, biodegradability, porosity and mechanical strength and so forth are the main characteristics that make hydrogel a potential candidate for the delivery of various therapeutic agents compared to other carrier systems [11]. Sodium alginate is a polysaccharide polymer produced either by bacteria or obtained from marine algae by the extraction process. It is hydrophilic, anionic, natural, abundant, nontoxic, biodegradable and biocompatible macromolecule composed of α-1, 4-L-glucuronic acid (G units) and poly-b-1, 4-Dmannuronic acid (M units) in various compositions by 1–4 linkages. Because of degree of swelling, rapid drug release and biodegradation rates under physiological conditions, sodium alginate is considered to be a suitable candidate for the loading and delivery of dyes, proteins, cells and drugs [12]. Thus, much attention has recently been given to sodium alginate in medical and industrial fields [13]. One ionic hydrophilic monomer which plays a vital role in synthesis of hydrogels for drug delivery is 2-acrylamido-2-methylpropanesulfonic acid (AMPS). Chondroitin sulfate based hydrogels were prepared by the free radical polymerization technique for the controlled release of loxoprofen where AMPS was used as monomer and exhibited maximum swelling and drug release in both acidic and basic media [14]. Similarly, chitosan based hydrogels were prepared by the polymerization technique. High drug loading and release was indicated by AMPS contents [15]. AMPS-based hydrogels exhibit swelling over the whole pH range because of the existence of strong ionizable sulfonic groups in its chain [3] that dissociate completely at any pH. Furthermore, an increase in the swelling index of hydrogel is observed as the concentration of AMPS increases, because of increase in the number of sulfonic group [16].

Here we report on SA-co-poly(AMPS) hydrogels for sustained release of DS to overcome the short comings associated with repeated dosing. A series of hydrogel formulations with various compositions of polymer, monomer and cross-linker were synthesized to assess and evaluate the different parameters including the sol–gel fraction, dynamic swelling and in-vitro drug release patterns of the developed hydrogel networks to confirm their sustained release properties for oral administration of DS.

## 2. Results and Discussions

### 2.1. Sol–Gel Analysis

**Figure 1:**
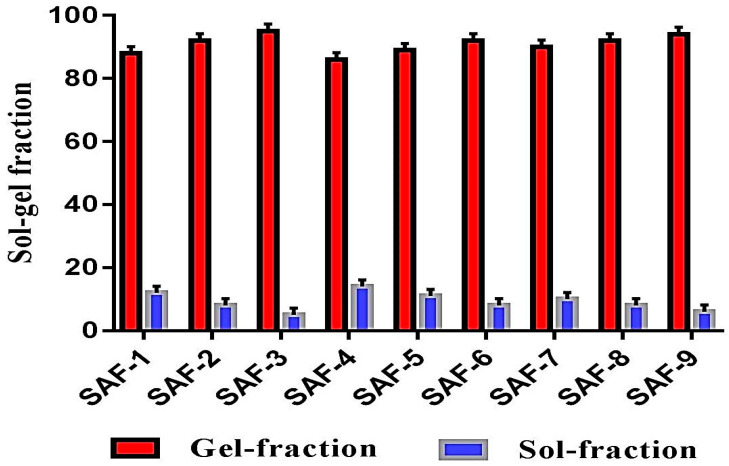
Sol–gel analysis of SA-co-poly(AMPS) hydrogels.

When polymer, monomer and crosslinker react during a polymerization reaction, some portion of the developed hydrogels is not crosslinked; this is known as the “sol fraction”, whereas the crosslinked portion is known as “gel fraction”. The sol portion is the small part of the hydrogel which may be formed due to the usage of high quantities of one or more than one component, and which remain uncrosslinked due to the unavailability of reactive sites during polymerization reaction. Thus, to know the fraction of crosslinked and uncrossliked portions of the hydrogels, sol–gel analysis was carried out for all fabricated formulations of hydrogel. Increase in gel fraction was observed as the concentration of SA, AMPS and MBA increases as shown in Figure 1. As the concentration of SA (SAF-1, SAF-2 and SAF-3), and AMPS (SAF-4, SAF-5 and SAF-6) increases, more space is provided for chemical reaction, and as a result the gel fraction is increased [17,18]. Similarly, as the concentration of MBA (SAF-7, SAF-8 and SAF-9) increases, cross-linking density of developed hydrogels increases, and as a result, an increase in gel fraction is observed [19] (Figure 1). Unlike gel fraction, a decrease in sol fraction is observed as the composition of all formulations (SAF-1~SAF-9) increases because sol fraction is inversely proportional to gel fraction [20].

### 2.2. FTIR Analysis

**Figure 2:**
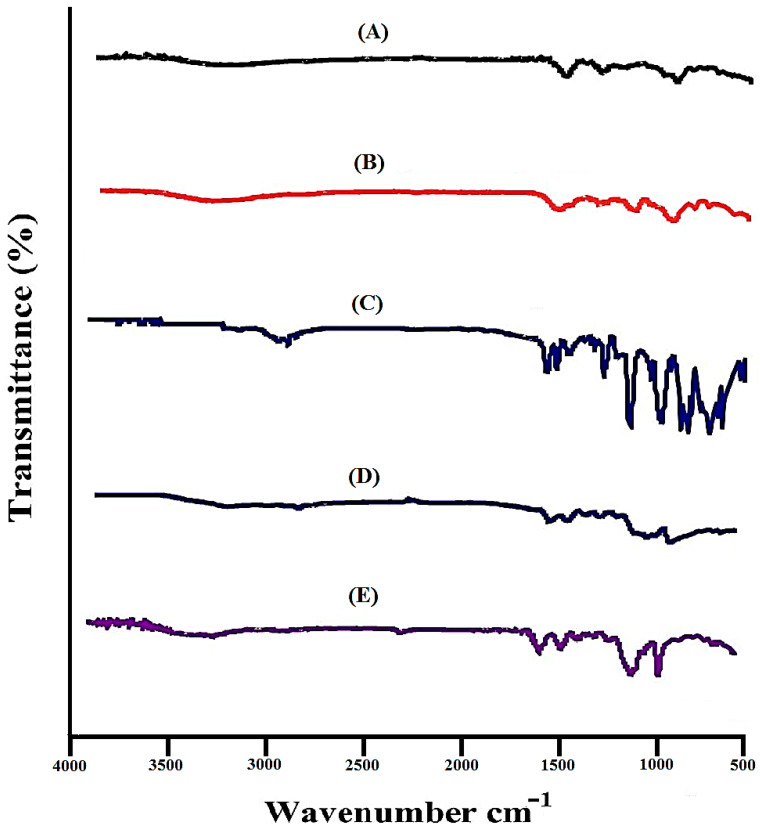
FTIR spectra of (**A**) DS, (**B**) SA, (**C**) AMPS, (**D**) unloaded SA-co-poly(AMPS) hydrogeI (**E**) loaded SA-co-poly(AMPS) hydrogel.

**Figure 3:**
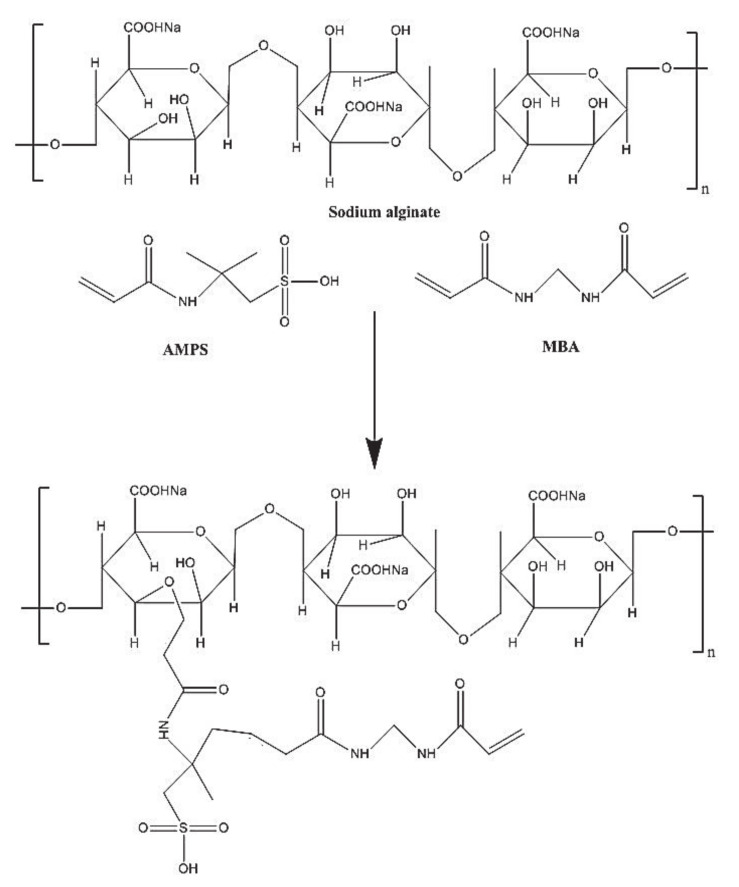
Possible chemical structure of SA-co-poly(AMPS) hydrogels.

FTIR was carried out to discover the structural arrangements of the contents used in the preparation of hydrogels individually and in the developed system. Figure 2A indicates FTIR spectrum of DS. COOH groups of DS indicate stretching vibrations at 3310 cm^−1^, while peaks at 3430 cm^−1^ and 1654 cm^−1^ characterize the stretching vibrations of N–H and C=C, respectively. Swain et al. (2015) reported the same peaks in their studies, which further support our studies [21]. The FTIR spectrum of SA (Figure 2B) indicates an extensive band at 3420 cm^−1^ due to OH groups of hydrogen bonding. Symmetric and asymmetric stretching vibrations of C=O of carboxyl groups of SA are indicated by peaks at 1405 cm^−1^ and 1570 cm^−1^, respectively. A characteristic peak present at 1105 cm^−1^ corresponds to –C–O–C stretching vibration of SA, indicating its polysaccharide structure [22]. The FTIR spectrum of AMPS is presented in Figure 2C. C–H stretching of methyl group is revealed by a sharp peak at 2998 cm^−1^. Bending and stretching of N–H and C=O are assigned by absorption bands at 1625 cm^−1^ and 1675 cm^−1^. Similarly, absorption bands at 1140 cm^−1^ and 1386 cm^−1^ indicate the symmetric and asymmetric stretching vibration of S=O group respectively [23]. Characteristic peaks at 3415 cm^−1^ and 2998 cm^−1^ of SA and AMPS are overlapped at 3350 cm^−1^ peak of unloaded SA-co-poly(AMPS) hydrogels as shown in Figure 2D. Similarly, SA and AMPS peaks at 1405 cm^−1^ and 1675 cm^−1^ are shifted to 1500 cm^−1^ and 1698 cm^−1^ in SA-co-poly(AMPS) hydrogels. While some peaks disappeared in the developed hydrogels. The shifting, disappearance, overlapping and formation of new peaks indicate the development of new polymeric network and demonstrate that AMPS is successfully grafted over the SA backbone [24]. Figure 2E indicates the FTIR spectrum of loaded SA-co-poly(AMPS) hydrogels. Some modified peaks of the drug are indicated in loaded SA-co-poly(AMPS) hydrogels at (2850 cm^−1^ and 3400 cm^−1^), which indicates that developed hydrogels have successfully encapsulated the drug. The proposed chemical structure of developed hydrogels is shown in Figure 3. No chemical interaction is found between drug and fabricated hydrogels. Hence the results indicate that hydrogels are successfully prepared by the crosslinking of both polymer and monomer and drug is loaded by the hydrogels without any type of interaction [25].

### 2.3. Thermal Stability

**Figure 4:**
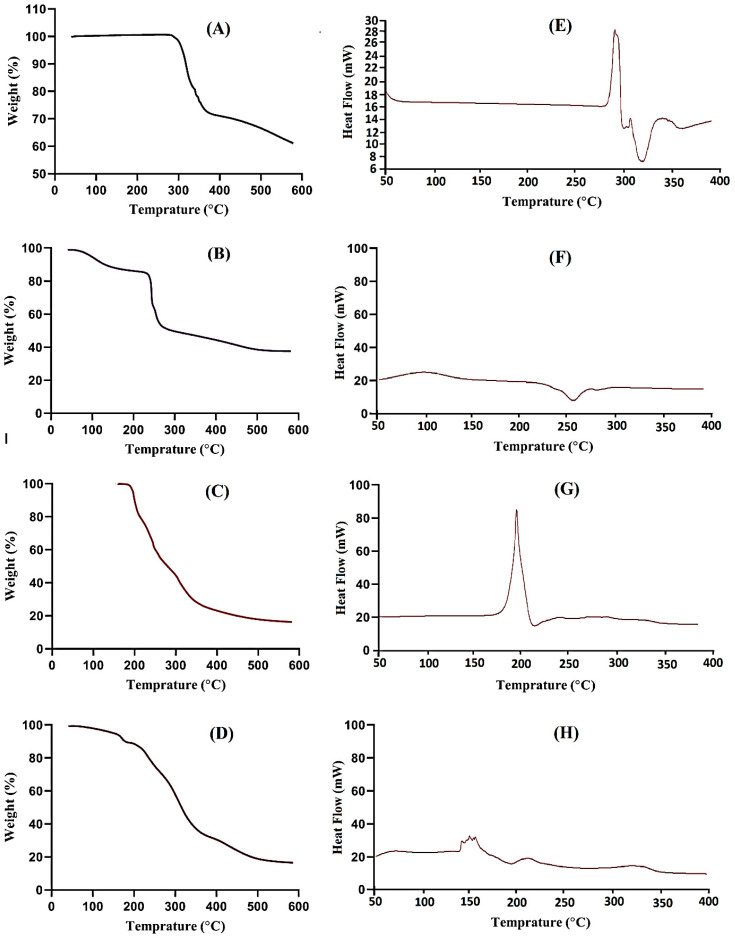
TGA of (**A**) DS, (**B**) SA, (**C**) AMPS (**D**) SA-co-poly(AMPS) hydrogelISC of (**E**) DS, (**F**) SA, (**G**) AMPS (**H**) SA-co-poly(AMPS) hydrogel.

Thermal stability was conducted to analyze the stability of the contents and developed system individually. Hence, TGA was accomplished for DS, SA, AMPS and SA-co-poly(AMPS) hydrogels as shown in Figure 4A–D. The TGA of DS reveals the loss of weight at three different steps (Figure 4A). The first step proceeds from 288 to 340 °C, and a 27% loss of weight is shown tracked by dehydration. At the second step, a 17% loss of weight is seen as the temperature approaches 443 °C. Finally, at the last step, drug pyrolysis stars from 457 °C and continues till completely paralyzed [26]. The TGA thermogram of SA (Figure 4B) indicates 13% weight loss from 77 to 188 °C, which relates to loss of water due to the segmental breakage of glucuronic acid and mannuronic acid with enhancement in temperature [27]. A subsequent weight reduction of approximately 33% starts from 215 °C up to 277 °C and accounts for a great rupture of the polymer backbone. Likewise, a weight loss of 12% is further observed within temperature range of 280–455 °C leading to minor damage to polymer chain [17]. The TGA thermogram of AMPS (Figure 4C) reveals weight loss of 5% up to 205 °C: further degradation of 42% is indicated within the temperature range from 202 to 225 °C. Similarly, the decomposition of the sulfonic acid group starts from 225 °C and continues till complete degradation [28]. The TGA thermogram of SA-co-poly(AMPS) hydrogels as shown in Figure 4D indicates that the degradation half-life of fabricated hydrogels (t_1/2_ = 450 °C) is greater than the degradation half-lives of its respective components i.e., SA (t_1/2_ = 320 °C) and AMPS (t_1/2_ = 320 °C) indicating high stability of the developed system. A 45% loss of weight is observed from 200 to 340 °C, followed by an additional 22% weight reduction within the temperature range of 340 to 400 °C due to the breakdown of carboxylate and sulfonate groups of polymers and monomers. Further degradation starts from 450 °C and continues till to complete paralysis of the developed system [29].

Figure 4E reveals the DSC of DS. Two endotherm peaks are observed at 282 °C and 328 °C correspondingly. Similarly at 287 °C and 342 °C, two exothermic peaks are shown. The first peak is assigned to the glass transition temperature, while the second peak specifies the degradation of drug [30]. The DSC thermogram of SA (Figure 4F) defines two endothermic peaks at 75 °C and 270 °C leading to the loss of moisture in hydrophilic groups of polymer and the rupturing of the backbone of the polymer chain, respectively. A higher intensity exothermic peak is found at 100 °C, leading to glass transition temperature Tg [31]. The DSC of AMPS is shown in Figure 4G indicating an endothermic peak at 180 °C and also indicating dehydration, whereas an exothermic peak at 75 °C reveals the glass transition temperature [28]. Moreover, another exothermic peak is detected at 198 °C revealing the AMPS degradation. The DSC thermogram of SA-co-poly(AMPS) hydrogels (Figure 4H) specifies two endothermic peaks at 140 °C and 325 °C. The endothermic peak of the polymer is moved from 270 °C to 325 °C in SA-co-poly(AMPS) hydrogels, which indicates the high stability of the developed hydrogels [17]. The results show that the developed hydrogel system is more thermally stable than its contents, which means that polymer, monomer and crosslinker polymerized successfully and developed a stable hydrogel network suitable for the sustained delivery of the drug.

### 2.4. SEM Analysis

**Figure 5:**
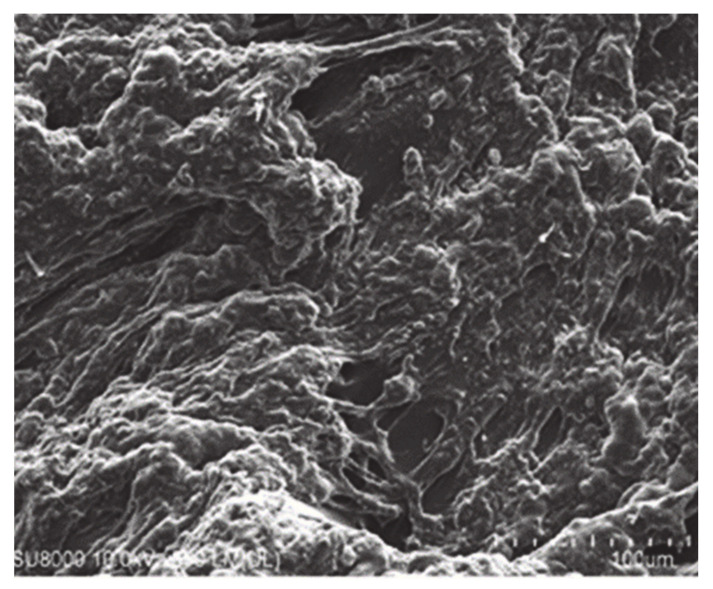
Surface morphology of SA-co-poly(AMPS) hydrogels.

SEM is carried out in order to understand the microstructure and surface morphology of hydrogels. Figure 5 indicates the uneven structure of hydrogels, which delivers a good space for retention of water, accommodation of the drug and the loading of solutes [32]. The surface morphology of different formulations is different. Small pores existing on the hydrogel’s surface influence the release of drug because of higher porosity and swelling. The higher swelling is due to the presence of COOH groups and sulfonate groups of SA and AMPS [33]. The pores form on the surface of the hydrogel is because of water evaporation by the heat medium of the reaction [34].

### 2.5. Analysis of PXRD

**Figure 6:**
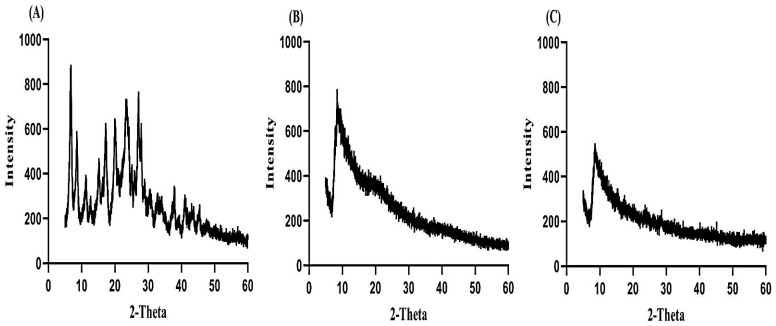
XRD of (**A**) DS, (**B**) unloaded SA-co-poly(AMPS) hydrogel, (**C**) loaded SA-co-poly(AMPS) hydrogel.

PXRD is conducted for the purpose of analyzing the crystallinity of the drug. Therefore, an X-ray pattern of DS, unloaded and loaded SA-co-poly(AMPS) hydrogels is performed as presented in Figure 6A–C. The characteristic peaks of DS are demonstrated at 2*θ* = 12.01, 24.29, 28.64 and 34.76° representing crystalline nature of drug (Figure 6A). The amorphous nature of SA-co-poly(AMPS) hydrogels is shown in Figure 6B. The intensity of the characteristic peaks of DS is decreased in loaded SA-co-poly(AMPS) hydrogels (Figure 6C which reveals that the crystallinity of the drug is reduced and encapsulated successfully by the amorphous system of hydrogels [35].

### 2.6. Drug Loading

Drug loading for all formulations was carried out by two methods, as shown in Table 1. As the concentration of SA (SAF1, SAF-2, and SAF-3) enhances, an increase in drug loading is observed [36]. The density and viscosity of the polymeric system is increased by increasing the concentration of SA, which retains the maximum drug inside the network, and thus a high quantity of drug is entrapped [37]. Similarly, as the concentration of AMPS (SAF-4, SAF-5 and SAF-6) increases, an increase in drug loading is detected. Unlike SA and AMPS, as the concentration of MBA (SAF-7, SAF-8 and SAF-9) increases, a decrease in drug loading is observed. Thus, drug loading is directly related to the swelling of hydrogels. A higher degree of swelling leads to greater drug encapsulation and vice versa [38].

### 2.7. Dynamic Swelling Experiment

**Figure 7:**
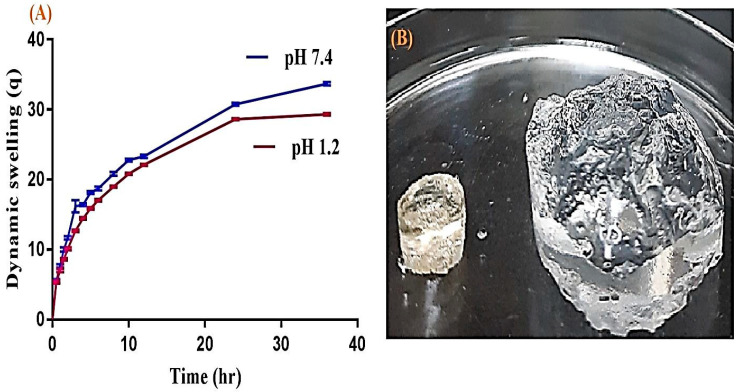
(**A**) Dynamic swelling of SA-co-poly(AMPS) hydrogels at pH 1.2 and pH 7.4, (**B**) dried and swelled form of SA-co-poly(AMPS) hydrogels.

The swelling study was carried out in order to understand the water/medium holding capacity of hydrogels. The swelling index depends on the types of polymer, monomer and crosslinker used in the preparation of the hydrogels. Therefore, a swelling study was performed for all SA-co-poly(AMPS) hydrogels at both pH 1.2 and pH 7.4 as indicated in Table 2, respectively. Slightly greater swelling is perceived at higher pH (7.4) as compared to lower pH (1.2) as indicated in Figure 7A. The dried and swelled form of hydrogel is indicated in Figure 7B. The reason is the higher concentration of COOH groups of SA at higher pH (7.4), due to which swelling in the basic medium is slightly higher than in the acidic medium [39,40]. Similarly to the concentration of SA (SAF1, SAF-2 and SAF-3) increasing at both pH 1.2 and pH 7.4, an increase in COOH groups occurs, which results in increased swelling [41,42] as shown in Table 2. As the concentration of SA increases, the pore size of the gel may be enlarged, which results in an enhancement in the penetration of water, thus high swelling is exhibited [35]. AMPS contains both ionic and nonionic groups. AMPS-based hydrogels start to exhibit higher swelling and become superabsorbent as the concentration of ionic groups increases. The higher swelling property of AMPS-based hydrogels is because of sulfonic groups. AMPS dissociates at various pH ranges with pH-independent swelling behavior. Similarly to the concentration of AMPS (SAF-4, SAF-5 and SAF-6) increases, the swelling behavior increases (Table 2) due to increase in ionic groups, which results in higher swelling [43,44]. Similar results of AMPS swelling have been reported by Kacmaz and Gurdag (2006) and Pourjavadi, Hosseinzadeh, and Mazidi (2005) [43,45]. In contrast to SA and AMPS swelling, the reduction in the swelling of the developed hydrogels is observed as the concentration of MBA (SAF-7, SAF-8 and SAF-9) increases (Table 2). The reason is the high crosslinking density of MBA, which increases as the composition of MBA increases; the hydrogels become hard and retard swelling. The compatible (three dimensional) structure of the hydrogel is because of the crosslinker. When a high concentration of the crosslinker is used, then crosslinking density is increased, or the pore size of the hydrogels decreases and, as a result, swelling reduces, which leads to reduction in drug loading and release (SAF-7,8 and 9) [46,47]. Figure 7B indicates the dried and swelled form of hydrogels. The dried form is transformed to swelled form once the hydrogel disc is immersed in any medium.

### 2.8. In Vitro Drug Release

#### 2.8.1. Influence of pH and SA, AMPS and MBA on Drug Release

**Figure 8:**
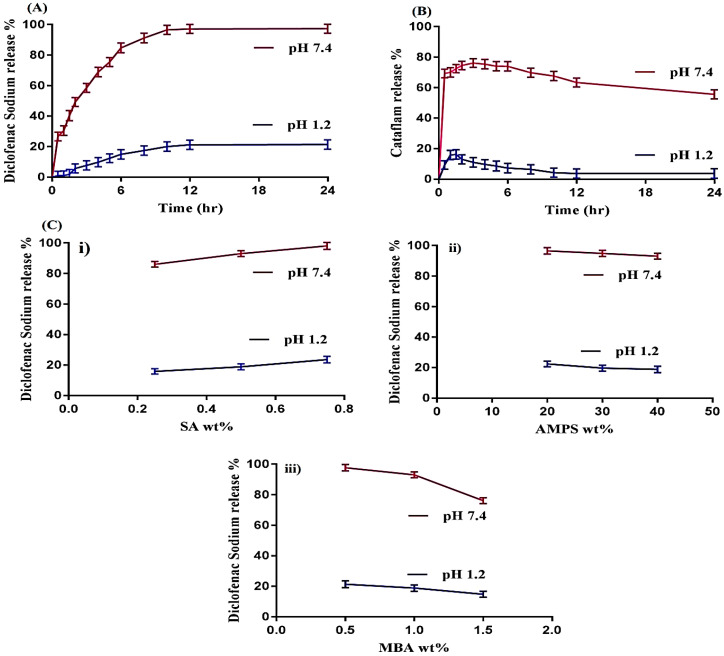
(**A**) Effect of pH on DS release percent from SA-co-poly(AMPS) hydrogels, (**B**) Effect of pH on release percent of commercial product Cataflam, and (**C**) Effect of i—SA, ii—AMPS and iii—MBA contents on DS release percent from SA-co-poly(AMPS) hydrogels at both pH 1.2 and 7.4.

An in vitro drug release study was performed to discover the percent of the drug released from the fabricated hydrogels at regular intervals of time. Hence, an in vitro release drug study was conducted for Cataflam (a marketed available dosage form of DS) and all SA-co-poly(AMPS) hydrogel formulations at both pH 1.2 and pH 7.4, as shown in Figure 8A–C. pH highly influences the percent of the drug released from the developed hydrogels, as greater drug release is observed in phosphate buffer pH 7.4 as compared to pH 1.2 (Figure 8A). The high percentage of release of drug at the higher pH 7.4 is because of the greater swelling of the hydrogels due to the increased number of deprotonated COOH groups as pH increases from lower to higher level. The number of COOH groups remains the same but the COOH groups that protonated in pH 1.2 to various other counter ions become deprotonated as the pH of the medium increases, due to which swelling increases and as result drug loading and the percentage of release of drug is increased. At pH 1.2, COOH groups attach to other groups and no free COOH group is available, whereas at pH 7.4 a number of deprotonated COOH groups are available, which repel each other and, as a result, swelling, drug loading and release increase. Similarly, the in vitro release of Cataflam is carried out in both media i.e., pH 1.2 and 7.4, respectively. A high amount the of drug (76%) is released within 2–3 h at pH 7.4, whereas at pH 1.2 a small amount (15%) is observed for initial 1–2 h (Figure 8B). After that a decline in the drug release rate is detected at both pHs. The percentage of drug released increases as the composition of SA increases (Figure 8C(i)) by keeping the constant composition of AMPS and MBA [48]. Contrary to SA, as the composition of AMPS increases, a decline in the percentage of drug release is detected (Figure 8C(ii)). The possible reason may be the interaction of AMPS with the drug [49,50]. The results indicate that as the composition of AMPS increases, an increase in swelling is observed due to the super swelling behavior of AMPS, but a decrease in drug release is detected at the same time. AMPS retards the drug to some extent due to its high concentration. The same behavior of AMPS is also reported by Saikia et al. [50] which further supports our study. Like AMPS, a decrease in the percentage of drug released is observed as the composition of MBA increases (Figure 8C(iii)). A tight junction is formed in polymeric network due to high composition of MBA, resulting in a reduction in pore size and a swelling of hydrogels, hence the percentage of drug released is decreased [34,51,52]. Hence, from the above discussion it could be concluded that the developed system could be used as a suitable carrier for the oral administration of DS.

#### 2.8.2. Kinetic Modeling

Kinetic modeling was performed for all formulations in order to deduce the drug release mechanism from developed SA-co-poly(AMPS) hydrogels as shown in Table 3. A suitable model was chosen on the basis of the closeness of the “*r*” value to 1. The “*r*” value is the regression coefficient. The “*r*” values of all models were compared. Hence, “*r*” values of zero order are in the range of 0.8886–0.9678, whereas for first order “*r*” values are in the range of 0.9733–0.9912. Similarly, for Higuchi and Korsmeyer–Peppas “*r*” values are in the range of 0.9656–9870 and 0.9611–0.9841, respectively. These all indicate that the “*r*” value for first order is higher than that for the zero order, Higchi and Korsmeyer–Peppas models, which mean that all formulations of the developed hydrogels exhibit first order drug release. The release data also show a good fit with Korsmeyer–Peppas model. The release exponent “*n*” value specifies the type of diffusion process, and “*n*” values for the developed system of hydrogels are in range of 0.4660–0.5430 (Table 3) confirming non-Fickian diffusion [53].

## 3. Conclusions

SA-co-poly(AMPS) hydrogels were prepared effectively by the crosslinking of natural polymer SA with the monomer AMPS in the presence of the cross-linker MBA, using APS as the initiator. The developed hydrogels showed pH-independent swelling and slightly greater swelling was exhibited at pH 7.4 as compared to pH 1.2 respectively. Swelling index was enhanced at both pH 1.2 and 7.4 as the concentration of SA and AMPS increased but decreased with increase in MBA concentration. Similarly like swelling study, drug loading was increased as the composition of polymer and monomer was increased while decreased with increase in MBA composition. The percentage of drug released was found higher at pH 7.4 as compared to pH 1.2, thus we can suggest that this system could be used for pH-dependent release of drug. TGA and DSC indicated that the developed system was thermally more stable than its contents. The porous structure was analyzed by scanning electron microscopy that revealed higher swelling. FTIR confirmed the successful structural arrangement of the monomer over the polymer backbone. PXRD analysis revealed that the crystalline drug was successfully loaded by the amorphous network of hydrogels, and also that the crystallinity of the drug was reduced. Conclusively, we can report that SA-co-poly(AMPS) hydrogels could be applicable for the sustained delivery of aqueous soluble drugs.

## 4. Material and Methods

### 4.1. Materials

DS was obtained from Alfa Aesar, Ward Hillm, MA, USA. SA was purchased from Acros (organics), Morris Plains, NJ, USA. Similarly, AMPS and MBA were obtained from Alfa Aesar, Lancashire, UK, respectively. Ammonium peroxodisulfate (APS) was acquired from Showa, Tokyo, Japan.

### 4.2. Development of SA-co-poly(AMPS) Hydrogels

In current research work, a series of formulations were developed by using various feed compositions of sodium alginate (SA), 2-acrylamido-2-methyl propane sulphonic acid (AMPS) and N,N′-Methylene bisacrylamide (MBA), respectively, as shown in Table 4. Sodium alginate-co-poly(2-acrylamido-2-methyl propane sulphonic acid) (SA-co-poly(AMPS)) hydrogels were developed through free radical polymerization technique. A precise quantity of SA was taken and dissolved in a precise volume of distilled water under a constant stirring. AMPS and ammonium peroxodisulfate (APS) solution were prepared separately in a required amount of distilled water. MBA was dissolved in mixture of ethanol and distilled water at 50 °C under constant stirring. Solution of APS was added slowly into AMPS solution. After a few minutes, mixture of AMPS and APS was added gradually into SA solution, followed by addition of MBA solution into SA solution. A translucent solution was formed after a proper mixing. The clear solution was transferred into glass tubes. Glass tubes were placed in water bath at 55 °C for 2 h, and then 65 °C for next 18 h respectively. The prepared gel was cut into discs of 5 mm and 8 mm, respectively. A 50% (*v*/*v*) mixture of water and ethanol was used to wash the discs of prepared gel in order to eradicate any unreacted gel contents. Discs of gel were dried at 25 °C for 2–4 h, and then kept in vacuum oven at 40 °C for 7 days to make the discs completely dried. The prepared hydrogel discs were used for analysis of various studies.

### 4.3. Characterization Determination

#### 4.3.1. Sol–Gel Analysis

Sol–gel fraction was carried out for all developed hydrogels formulations in order to eradicate any unreacted content. Gel is the gelling part of crosslinked hydrogel contents, whereas sol is the soluble unreacted part of developed hydrogels. Hydrogel discs (Q_1_) of specific size were selected for soxhlet extraction process, and kept in deionized distilled water at 85 °C for 9 h. The extracted discs of hydrogels (Q_2_) were put in an oven at 40 °C for drying till the discs dried completely. Sol–gel fraction was analyzed by using given Equations (1) and (2) respectively [54].

(1) $$\mathrm{Sol}\;\mathrm{fraction}\;\%=\frac{{\;Q}_{1}-{\;Q}_{2}}{{\;Q}_{2}}\times 100$$

where, Q_1_ = Initial weight of hydrogels disc, and Q_2_ = final weight of hydrogel disc.

(2) Gel fraction=100−Sol fraction

#### 4.3.2. Thermal Stability

Thermogravimetric analysis (TGA) (Simultaneous Thermal Analyzer STA 8000, PerkinElmer, Waltham, MA, USA) was conducted for DS, SA, AMPS and developed hydrogels. TGA was conducted to examine the thermal stability of all samples. The samples were analyzed beneath constant flow of nitrogen. Heat was maintained between 20 and 400 °C for all samples. Similarly, differential scanning calorimetry (DSC) (PerkinElmer DSC 4000, Waltham, MA, USA) was performed for all samples as mentioned above. Nitrogen flow was kept constant throughout the study with heating rate of 20 °C/min [55].

#### 4.3.3. Scanning Electron Microscopy (SEM) Study

JSM-5300 model (Jeol, Tokyo, Japan) of SEM was processed to analyze the surface morphology of fabricated hydrogels. Scanning of developed hydrogels was carried out at different magnifications [56].

#### 4.3.4. FTIR Analysis

Analysis of FTIR was done for drug, SA, AMPS, unloaded SA-co-poly(AMPS) hydrogels and drug loaded SA-co-poly(AMPS) hydrogels. Pestle and mortar were used to crush all samples to desired particle size carefully and then NICOLET 380 FTIR (Thermo Fisher Scientific, Ishioka, Japan) was used for analysis in range of 4000–500 cm^−1^ [57].

#### 4.3.5. Analysis of PXRD

Powder X-ray diffractometry (PXRD) (XRD-6000 SHIMADZU, Tokyo, Japan) was conducted for drug, unloaded hydrogels and drug loaded hydrogels at room temperature. All samples were griped by plastic sample holder and surface was smoothed by glass slide. Range of theta (θ) was maintained between 10 and 60° at a rate of 2° 2θ/min for sample analysis [55].

### 4.4. Loading of Diclofenac Sodium

Absorption method was used for drug loading. A drug solution of 1% *w*/*v* was prepared in phosphate solution of buffer (pH 7.4). Weighed dried hydrogel discs were placed in drug solution for 72 h. After this, the hydrogel discs were taken out, blotted with filter paper and placed in a vacuum oven at 40 °C till the discs dried completely [54,58]. Two techniques were reported for quantification of drug load in hydrogel discs. First technique was solvent replacement method where loaded discs of hydrogel were submerged in 25 mL of fresh buffer solution (pH 7.4) overnight. Buffer was replaced by fresh buffer and evaluated by UV–vis-spectrophotometer (U-5100, 3J2-0014, Tokyo, Japan) at wavelength of 260 nm. This process was repeated until the entire drug was eliminated completely from the hydrogel discs. Another technique is dry weight method. In this technique, the quantity of loaded drug was quantified by immersing the weighed dried discs of hydrogel in drug solution, dried in oven at 40 °C and weighed again. Quantification of drug was calculated by the difference in weight of hydrogel discs after immersion in drug solution once dried completely and before immersion in drug solution. Loading of drug is estimated by given Equation (3) [59]:

amount of Drug loaded = W_D_ − W_d_ (3)

where, W_D_ = weight of dried loaded disc of hydrogel, and W_d_ = weight of dried unloaded disc of hydrogel.

### 4.5. Swelling Experiment

Dynamic swelling was conducted at both pH 1.2 and pH 7.4 respectively. Weighed dried hydrogel discs (S_1_) were placed in freshly prepared buffer solutions of pH 1.2 and pH 7.4 of 100 mL. After a regular time interval, the discs were taken out and blotted with a filter paper and weighed again (S_2_). This process was continued till an equilibrium swelling was accomplished. Equations (4) and (5) were used to calculate dynamic and equilibrium swelling, respectively [60].

(4) $$q=\frac{{\;S}_{2}}{{\;S}_{1}}\;$$

where; q = dynamic swelling, S_1=_ initial weight before swelling, and S_2=_ final weight after swelling at time t.

(5) $$\mathrm{SR}\%=\frac{{\;P}_{1}-{\;P}_{2}}{P_{2}}\times 100\;$$

where; SR = Swelling ratio, P_1_ = weight of swollen hydrogel discs, while P_2_ = weight of dry hydrogel discs.

### 4.6. In Vitro Study

An in vitro drug study was conducted for marketed dosage form of DS (Cataflam) and all formulations of fabricated hydrogels at both pH 1.2 and pH 7.4, respectively. The hydrogel loaded discs were placed in the oven at 40 °C till equilibrium weight was attained and then assessed for dissolution test. The dissolution media were maintained 900 mL at 50 rpm and 37 ± 0.5 °C for 24 h throughout the in vitro study. A sample was taken at specific time intervals and then analyzed by UV–vis-spectrophotometer (U-5100, 3J2-0014, Tokyo, Japan) at wavelength of 260 nm. The same quantity of fresh buffer was added into the dissolution medium as that which was taken in order to maintain a sink condition [61].

Kinetic modeling of Zero order, First order, Higuchi model and Korsmeyer−Peppas model were carried out for all developed hydrogels in order to determine the mechanism of drug release [62].

## Figures and Tables

**Table 1:** Drug loading of SA-co-poly(AMPS) hydrogel.

Formulation	Amount of DS Loaded in Hydrogels (mg)/450 mg of Dry Gel	
Code	Extraction Method	Weight Method
SAF-1	87.13 ± 1.03	88.45 ± 0.99
SAF-2	92.97 ± 0.91	93.02 ± 0.82
SAF-3	98.52 ± 0.83	99.08 ± 0.12
SAF-4	89.15 ± 0.76	88.72 ± 0.51
SAF-5	91.10 ± 0.81	91.36 ± 0.92
SAF-6	92.97 ± 0.91	93.02 ± 0.82
SAF-7	97.10 ± 1.06	96.43 ± 0.93
SAF-8	92.97 ± 0.91	93.02 ± 0.82
SAF-9	75.48 ± 1.03	74.78 ± 0.95

**Table 2:** Dynamic swelling of SA-co-poly(AMPS) hydrogel.

Formulation	Dynamic Swelling up to 36 h	
Code	pH 1.2	pH 7.4
SAF-1	19.82 ± 0.07	20.82 ± 0.21
SAF-2	20.36 ± 0.20	21.85 ± 0.19
SAF-3	21.01 ± 0.15	22.20 ± 0.12
SAF-4	12.24 ± 0.17	16.02 ± 0.22
SAF-5	16.04 ± 0.14	18.02 ± 0.12
SAF-6	20.36 ± 0.20	21.85 ± 0.19
SAF-7	29.33 ± 0.16	33.68 ± 0.24
SAF-8	20.36 ± 0.20	21.85 ± 0.19
SAF-9	17.54 ± 0.13	18.06 ± 0.16

**Table 3:** Kinetic modeling release of DS from SA-co-poly(AMPS) hydrogels.

Formulae	Zero Order	First Order	Higuchi	Korsmeyer-Peppas	
Code	*r* ^2^	*r* ^2^	*r* ^2^	*r* ^2^	*N*
SAF-1	0.9678	0.9865	0.9772	0.9649	0.5319
SAF-2	0.9374	0.9912	0.9806	0.9611	0.4945
SAF-3	0.8886	0.9854	0.9656	0.9710	0.4660
SAF-4	0.9461	0.9733	0.9858	0.9777	0.4923
SAF-5	0.9497	0.9898	0.9853	0.9720	0.4999
SAF-6	0.9374	0.9912	0.9806	0.9611	0.4945
SAF-7	0.9293	0.9857	0.9870	0.9841	0.4677
SAF-8	0.9374	0.9912	0.9806	0.9611	0.4945
SAF-9	0.9908	0.9833	0.9792	0.9754	0.5430

**Table 4:** Various compositions of SA-co-poly(AMPS) hydrogel formulation.

Formulation Code	Polymer Sodium Alginate (g/100 g)	Monomer AMPS (g/100 g)	Cross-Linker MBA (g/100 g)	Initiator APS (g/100 g)
SAF-1	0.25	40	1.0	0.5
SAF-2	0.50	40	1.0	0.5
SAF-3	0.75	40	1.0	0.5
SAF-4	0.50	20	1.0	0.5
SAF-5	0.50	30	1.0	0.5
SAF-6	0.50	40	1.0	0.5
SAF-7	0.50	40	0.5	0.5
SAF-8	0.50	40	1.0	0.5
SAF-9	0.50	40	1.5	0.5

AMPS: 2-acrylamido-2-methyl propane sulphonic acid. MBA: N,N′-Methylene bisacrylamide. APS: Ammonium peroxodisulfate.
